# Gender-specific effects of HIV protease inhibitors on body mass in mice

**DOI:** 10.1186/1742-6405-4-8

**Published:** 2007-05-01

**Authors:** Melinda E Wilson, Kimberly F Allred, Elizabeth M Kordik, Deana K Jasper, Amanda N Rosewell, Anthony J Bisotti

**Affiliations:** 1 Department of Physiology, College of Medicine, University of Kentucky, Lexington, KY 40536, USA

## Abstract

Protease inhibitors, as part of highly active anti-retroviral therapy (HAART), have significantly increased the lifespan of human immunodeficiency virus (HIV) infected patients. Several deleterious side effects including dyslipidemia and lipodystrophy, however, have been observed with HAART. Women are at a higher risk of developing adipose tissue alterations and these alterations have different characteristics as compared to men. We have previously demonstrated that in mice the HIV protease inhibitor, ritonavir, caused a reduction in weight gain in females, but had no effect on male mice. In the present study, we examined the potential causes of this difference in weight gain. Low-density lipoprotein receptor (LDL-R) null mice or wild-type C57BL/6 mice, were administered 15 μg/ml ritonavir or vehicle (0.01% ethanol) in the drinking water for 6 weeks. The percent of total body weight gained during the treatment period was measured and confirmed that female LDL-R gained significantly less weight with ritonavir treatment than males. In wild type mice, however, there was no effect of ritonavir treatment in either sex. Despite the weight loss in LDL-R null mice, ritonavir increased food intake, but no difference was observed in gonadal fat weight. Serum leptin levels were significantly lower in females. Ritonavir further suppressed leptin levels in (p < 0.05). Ritonavir did not alter serum adiponectin levels in either gender. To determine the source of these differences, female mice were ovariectomized remove the gonadal sex hormones. Ovariectomy prevented the weight loss induced by ritonavir (p < 0.05). Furthermore, leptin levels were no longer suppressed by ritonavir (p < 0.05). This study demonstrates that gonadal factors in females influence the hormonal control of weight gain changes induced by HIV protease inhibitors in an environment of elevated cholesterol.

## Background

The use of highly active anti-retroviral therapy (HAART) has dramatically increased the lifespan of individuals infected with the human immunodeficiency virus (HIV). HAART often includes a cocktail of nucleoside reverse transcriptase inhibitors and protease inhibitors that prevent virus replication and assembly. While effective in reducing the progression of AIDS, significant side effects have been observed with long-term use of protease inhibitors [1-3]. HIV protease inhibitors have been associated with an increase in atherosclerosis, dyslipidemia and lipodystrophy. Adipose tissue alterations associated with protease inhibitor use include a loss of total body fat, with an increase in fat deposition in the abdomen and in the dorsocervical region leading to "buffalo humps" [4-6]. This pattern of fat distribution is often associated with the complex of symptoms including insulin resistance, hypertension and dyslipidemia referred to as metabolic syndrome [7].

Gender differences have been observed in the incidence as well as the severity of these adipose tissue alterations, with women having a higher rate of reported disturbances [8]. The adipose tissue alterations observed were complex and result in increased abdominal and breast accumulation with reduced peripheral fat. These differences were not due to age or the severity of the disease and are hypothesized to be hormonal in nature.

Several adipose tissue-derived hormones play a role in weight gain, obesity and are involved in the development of metabolic syndrome [9-11]. Leptin plays a crucial role for regulating weight gain by controlling fat mass. Leptin levels are positively correlated with body mass index [12]. Additionally, leptin has been shown to reverse the dyslipidemia and lipodystrophy caused by HIV protease inhibitors in mice [13]. Adiponectin is also produced from adipose tissue and sensitizes skeletal muscle and liver to the actions of insulin [14]. Adiponectin levels are negatively correlated with body mass index [15].

Ritonavir induces atherosclerotic lesions in low-density lipoprotein receptor knockout (LDL-R null) mice [16]. We previously observed that females gained significantly less weight than their male counterparts [17]. In the present study, we have begun to investigate possible mechanisms of this gender difference in male and female mice undergoing treatment with the HIV protease inhibitor, ritonavir.

## Results

### Ritonavir treatment reduced weight gain in female LDL-R null mice

**Figure 1:**
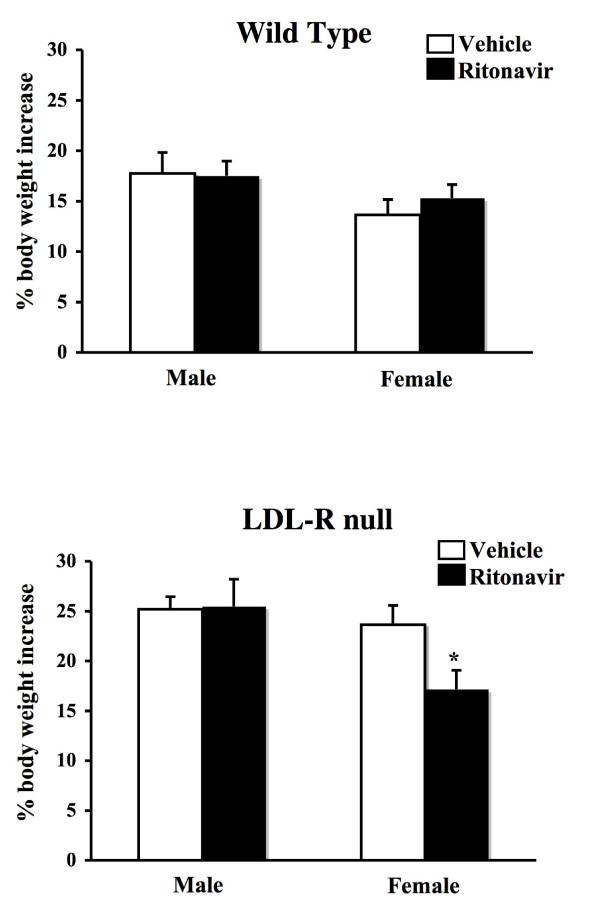
**Weight gain in mice treated with ritonavir**. At six weeks of age, male and female wild type (C57BL/6) (top) or LDL-R null mice (bottom) were administered ritonavir (15 μg/day) or vehicle (0.01% ethanol) through the drinking water for six weeks. Mice were weighed at the beginning and at the conclusion of the study. Bars represent the mean +/- SEM, n = 6–8. * = significantly different from vehicle (p < 0.05).

We had previously observed a decrease in weight gain in female LDL-R null mice receiving ritonavir in the drinking water as compared to males [17]. To begin to explore the mechanisms of this effect in more detail we addressed the effect of ritonavir treatment in male and female wild type (C57BL/6) as well as LDL-R null mice. Both genotypes were used to determine if the elevated cholesterol associated with LDL-R null mice [17], played a role in weight gain. Beginning at six weeks of age male and female wild type (C57BL/6) and LDL-R null mice were weighed and randomly assigned to two treatment groups. One group received vehicle (0.01% ethanol), while the other received ritonavir (15 μg/day) in the drinking water as previously described [16]. At the end of 6 weeks of treatment, the animals were weighed again. In wild type mice, both males and females gained the same amount of weight expressed as a percentage of total body weight (Figure 1). Ritonavir had no effect on weight gain. LDL-R null mice gained more weight overall. Ritonavir had no effect in males, but suppressed weight gain in females (p < 0.05).

### Ritonavir treatment does not alter serum levels of cholesterol, insulin, glucose or 17β-estradiol

We have previously utilized this paradigm to investigate the ability of ritonavir to induce atherosclerosis without raising cholesterol levels further to isolate the direct effects of ritonavir. LDL-R null mice had elevated cholesterol compared to wild type mice, but ritonavir had no further effect (Table 1). We confirmed that this dose of ritonavir did not alter serum cholesterol levels. Ritonavir treatment did not alter levels of insulin, blood glucose or 17β-estradiol in female mice.

**Table 1:** Metabolic parameters of LDL-R null mice treated with ritonavir.

	**Male**		**Female**	
	
	**Vehicle**	**Ritonavir**	**Vehicle**	**Ritonavir**
**Total Cholesterol (mg/ml)**	1.32 +/- 0.06	1.23 +/- 0.11	0.96 +/- 0.06*	0.98 +/- 0.08*
**Insulin (ng/ml)**	0.80 +/- 0.01	0.73 +/- 0.01	0.80 +/- 0.08	0.72 +/- 0.02
**Glucose (mg/dL)**	180.17 +/- 17.6	176.67 +/- 14.5	155.17 +/- 6.4	149.00 +/- 8.2
**Estradiol (pg/ml)**	ND	ND	9.19 +/- 1.8	12.75 +/- 2.3

* = significantly different from male mice (p < 0.05)

ND = Not Determined

### Ritonavir does not alter adipose tissue amount in females

**Figure 2:**
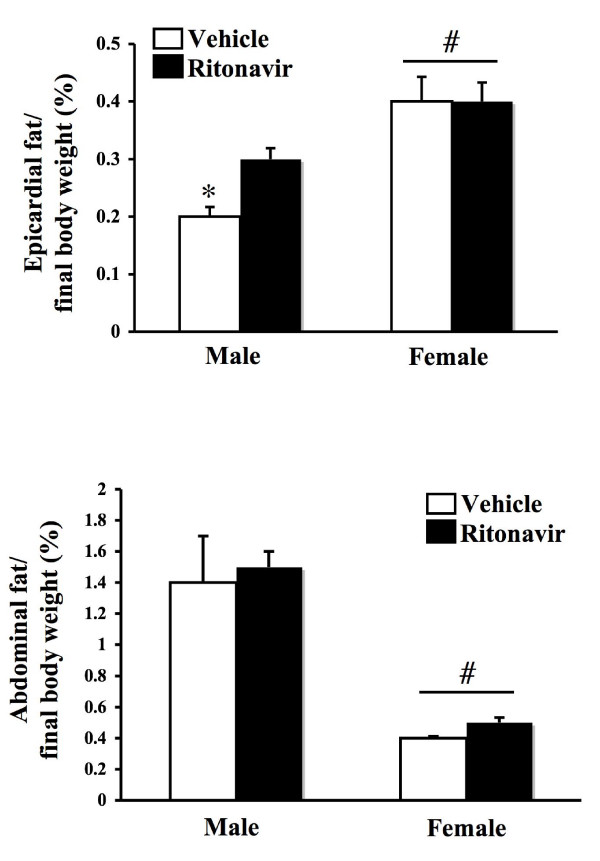
**Adipose tissue in mice treated with ritonavir**. Male and female LDL-R null mice were administered ritonavir (15 μg/day) or vehicle (0.01% ethanol) through the drinking water for six weeks. At the conclusion of the study, epicardial and abdominal adipose tissue was removed and weighed. Data is expressed as a percentage of final body weight. Bars represent the mean +/- SEM, n = 6–8. * = significantly different from ritonavir (p < 0.05). # = significantly different from males (p < 0.05).

Since we observed no effect of ritonavir in wild type mice, all future studies were performed in LDL-R null mice. Epicardial and abdominal fat are important sources of adipose tissue that play a role in the development of metabolic syndrome and cardiovascular disease [18]. As these two processes are associated with HAART, especially ritonavir treatment, epicardial fat was removed and weighed at the conclusion of the experiment. Epicardial fat was dissected from the heart and weighed (Figure 2A). Males had less epicardial fat, and ritonavir treatment raised it to levels approaching those in females (p < 0.05). Epicardial fat does not correlate with alterations in weight gain. Additionally we isolated white adipose tissue from the gonadal fat pad in the abdomen as previously described [19](Figure 2B). Females had significantly less abdominal fat adjusted for body weight as compared to males (p < 0.05). Ritonavir treatment did not alter abdominal fat in either sex.

### Ritonavir increases food intake

Mice were monitored during the six weeks of ritonavir treatment to assess their average daily water and food intake (Table 2). Ritonavir increased water intake in males (p < 0.05). No effect was seen in females. In both sexes, ritonavir increased food intake (p < 0.01).

**Table 2:** Food and water intake of LDL-R null mice treated with ritonavir.

	**Male**		**Female**	
	
	**Vehicle**	**Ritonavir**	**Vehicle**	**Ritonavir**
**Water intake (ml/day)**	2.78 +/- 0.084	3.42 +/- 0.140	2.83 +/- 0.091*	2.96 +/- 0.107*
**Food intake (g/day)**	3.21 +/- 0.047	6.05 +/- 0.017#	2.87 +/- 0.035*	5.48 +/- 0.062*#

* = significantly different from male mice (p < 0.05)

# = significantly different from vehicle (p < 0.05)

### Leptin levels were suppressed by ritonavir

**Figure 3:**
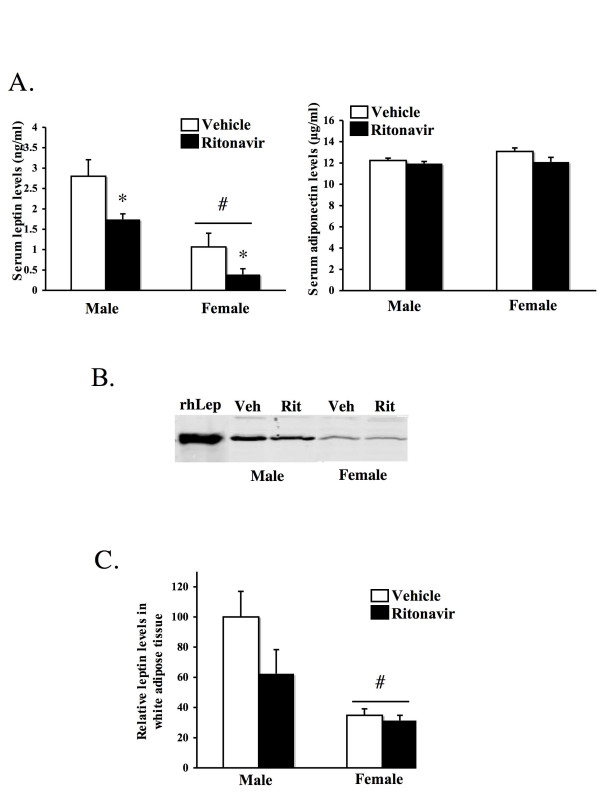
**Adipokine levels in mice treated with ritonavir**. **A) **Serum leptin levels (left) and adiponectin levels (right) were measured by ELISA in male and female LDL-R null mice treated with ritonavir (15 μg/day) or vehicle (0.01% ethanol) for six weeks. Bars represent the mean +/- SEM, n = 6–8. * = significantly different from vehicle (p < 0.05). # = significantly different from males (p < 0.05). **B) **Leptin expression was identified by western immunoblot assay. Total protein was isolated from white adipose tissue from male and female LDL-R null mice treated with ritonavir (15 μg/day) or vehicle (0.01% ethanol) for six weeks. SDS-PAGE and immunoblot with a leptin antibody (#AFP6621299 obtained through the NHPP, NIDDK and Dr. A. F. Parlow) was performed. The relative density of the bands was quantified. **C) **A representative immunoblot is shown. Recombinant human leptin (rhLeptin) protein was included as a positive control.

To begin to investigate potential hormonal mechanisms by which ritonavir modulates weight gain we measured the effect on two of the important adipose hormones involved in weight gain; leptin and adiponectin. At the end of the treatment period, leptin and adiponectin levels were measured in the serum. Females had lower serum levels of leptin as compared to males (p < 0.05) in either treatment group (Figure 3A). Ritonavir treatment reduced leptin levels in both males and females (p < 0.05). There was no effect of ritonavir on adiponectin levels (Figure 3A). Additionally, we measured leptin protein levels in white adipose tissue by western immunoblot analysis (Figure 3B). The relative density of leptin expression in adipose protein samples was quantified and expressed relative to vehicle treated males (Figure 3C). Blots were also processed with an antibody to actin to ensure equal protein levels in each lane (data not shown). The protein levels in the adipose tissue reflect the serum levels in terms of gender. Ritonavir, however, did not significantly alter leptin expression within white adipose tissue.

### Ovariectomy reverses the effects of ritonavir in female mice

**Figure 4:**
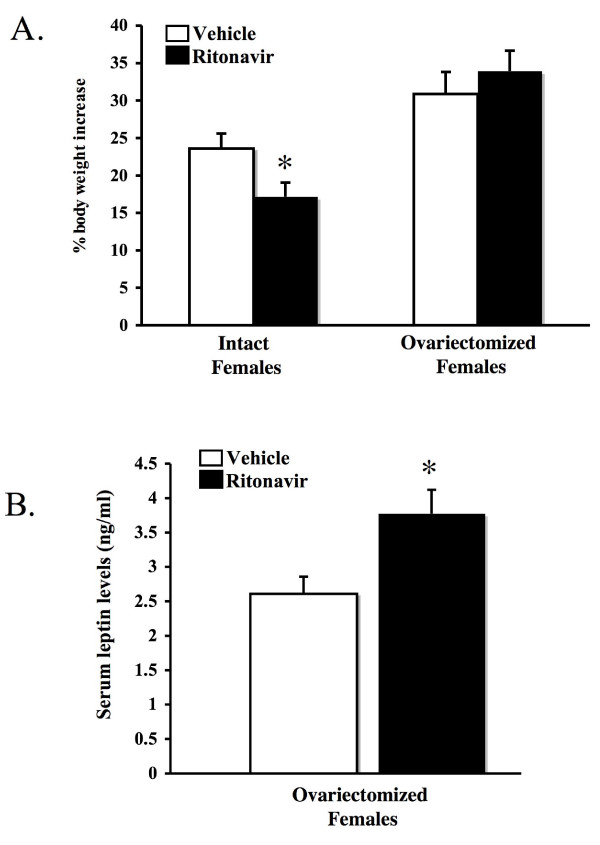
**Ovariectomy reverses the effect of ritonavir in female mice**. **A) **At six weeks of age, female LDL-R null mice were bilaterally ovariectomized and administered ritonavir (15 μg/day) or vehicle (0.01% ethanol) through the drinking water for six weeks. An intact group was included as a control. Mice were weighed at the beginning and at the conclusion of the study. Bars represent the mean +/- SEM, n = 6–8. **B) **Serum leptin levels were measured by ELISA in ovariectomized female mice treated with ritonavir (15 μg/day) or vehicle (0.01% ethanol) for six weeks. Bars represent the mean +/- SEM, n = 6–8. * = significantly different from vehicle (p < 0.05).

To determine if hormonal factors from the ovary contribute to the gender-specific effects of ritonavir on weight gain, the ovaries were surgically removed from female mice at the beginning of the ritonavir treatment period. Intact control animals lost weight as before (p < 0.05) (Figure 4A). Ovariectomy prevented the weigh loss induced by ritonavir. Ovariectomy also induced a small but statistically insignificant gain in weight. No significant differences were observed in the epicardial fat or the gonadal fat pat in these animals (data not shown). Serum leptin levels were also measured at the conclusion of the ritonavir treatment (Figure 4B). Ritonavir no longer suppressed leptin levels in ovariectomized mice. Leptin levels were significantly increased (p < 0.05).

## Discussion

In the present study, we have demonstrated that gender influences one of the side effects of HIV protease inhibitors by inducing different outcomes of weight gain in male and female mice. Ritonavir treatment suppressed body mass gain in female LDL-R null mice. Interestingly, this effect only occurred in LDL-R null mice and not wild type mice. Ritonavir also decreased leptin levels in serum while increasing food intake. Ovariectomy prevented the weight reduction and suppression of leptin in females, indicating gonadal factors mediate this alteration in weight gain in response to ritonavir.

We previously demonstrated that ritonavir treatment in female LDL-R null mice caused a decrease in body weight gain over the course of the six-week treatment period [17]. This dose of ritonavir does not have the same effect in wild type mice. Other studies with higher doses, however, have shown decreased weight gain in wild type mice [20] suggesting that there is a continuum of effects based on the dose of drug. The lower dose of ritonavir in this study is relevant as low doses are often used to boost the bioavailability of other components of HAART [21,22]. The primary difference between LDL-R null and wild type mice is elevated cholesterol and triglyceride levels. Elevated serum triglyceride levels are one factor involved in the metabolic consequences of HAART leading to cardiovascular disease and atherosclerosis [23]. The results of the current study suggest that these differences also makes females more susceptible to alterations in weight gain and adipose tissue formation induced by ritonavir treatment. The level of the molecular interaction of this effect remains to be determined.

Baseline leptin levels were lower in females. This has previously been shown in wild type CD-1 mice, but to our knowledge this is the first time it has been demonstrated in LDL-R null mice [24]. Other studies with C57BL/6 mice have shown no difference or small increases in leptin in females [25,26]. These differences are likely due to genetic background or the age of the mice. This difference was observed both in circulating levels of leptin in the serum as well as at the level of leptin expression in abdominal white adipose tissue.

In the present study, ritonavir suppressed serum leptin levels, and increased food intake in both male and female mice. This increase in food intake did not translate to increased abdominal fat deposition or weight gain. Interestingly, ritonavir increased epicardial fat in males. This may correlate with the increased susceptibility of males to the development of atherosclerosis in this model of HIV protease inhibitor treatment [17]. Alternatively, in females, weight gain is impaired. One possible explanation is that ritonavir causes an alteration in energy expenditure and metabolic activity of the liver or skeletal muscle. Ritonavir has been shown to alter gene expression in the liver that results in altered fatty acid metabolism [20]. Even though in the present study, ritonavir does not alter serum lipids, it can have subtle effects on liver and adipose metabolism.

Ritonavir suppressed leptin levels in both male and female mice. Leptin levels in females were lower than males to begin with. It is possible that there is a threshold level at which if leptin levels drop below, the ability to maintain body weight in the face of a metabolic challenge is lost. Previously, studies have shown that at high concentrations of ritonavir, leptin levels are reduced [13]. One possible explanation for the lack of correlation between tissue expression and serum levels is an alteration in leptin binding proteins by ritonavir. Ritonavir may regulate serum binding proteins or the soluble leptin receptor. These binding proteins affect the stability, deliverance of leptin to targets or its clearance rate [27].

Women are more likely to develop adipose tissue alterations induced by HAART [8]. Our data suggests that in mice this is also the case. Clinical studies have demonstrated that not all women develop adipose tissue alterations and that they do not manifest themselves in the same manner or have the same time of onset [8,28]. Elevated triglycerides are, however, associated with the development of adipose tissue alterations [28]. Our data add to the growing body of evidence that females who have dyslipidemia would be more likely to develop adipose tissue alterations. Additionally, it is also possible that gender influences the metabolism or bioavailability of ritonavir. This gender difference has been shown for the HIV protease inhibitor, indinavir [29].

Ovariectomy is well known to increase body weight in rodents and humans [30]. The exact role of estrogen in this process is not clear, but may involve regulation of leptin or alterations in lipid metabolism in skeletal muscle and adipose tissue [31,32]. We observed an increase in weight gain following ovariectomy. Ritonavir did not influence weight gain in the ovariectomized female mice, suggesting an interaction at the site of action of ritonavir and ovariectomy in the ability of the two to influence body mass. Whether this interaction involves gonadal hormones, such as estrogen, remains to be determined.

LDL-R null mice produce an environment of elevated plasma cholesterol and triglycerides. It is in this environment that ritonavir alters weight gain. It remains possible that the loss of LDL-R protein itself causes the effect. This possibility has not been previously studied in detail in these mice, but a lack of LDL-R in tissues other than the liver may be important in regulating body weight. Additionally, LDL-R is expressed at a relatively high level in the adrenal gland and could potentially play a role in regulating body weight by altering cortisol production [33].

## Conclusion

In conclusion, this study reports the novel finding that female mice are more likely to develop disturbances in weight gain in response to ritonavir when they have a background of elevated cholesterol. Ritonavir causes a decline in serum leptin levels without altering adiponectin levels. In concordance with the decline in leptin levels there was increased food intake, however, no difference in abdominal fat was observed. This suggests a secondary site of action where ritonavir prevents adipose tissue formation or and increase in energy expenditure. Removing the female sex hormones prevents the effects of ritonavir on weight gain and serum leptin levels. This study begins to investigate the mechanisms involved in the diverse actions of HIV protease inhibitors and underscores the complexity of the interactions between female hormones and metabolism.

## Methods

### Animals

All animals were housed in the AAALAC certified animal facilities at the University of Kentucky. Animals were maintained on a 14:10 light/dark cycle at constant temperature conditions with food (normal chow) and water provided ad libitum. Wild type C57BL/6 mice were purchased from Charles River (Wilmington, MA). The LDL-R null and mice were supplied by The Jackson Laboratory (Bar Harbor, ME). LDL-R null mice have been backcrossed to a C57BL/6 background. At six weeks of age mice were given vehicle control (0.01% ethanol) or ritonavir (15 μg/day) in their drinking water for 6 weeks. A stock ritonavir solution was made in ethanol and further diluted in the drinking water. This regimen has previously been described to induce atherosclerotic lesions in LDL-R null mice without further altering plasma cholesterol levels [16]. It produces significant physiological effects at relatively low doses of ritonavir. At the time of tissue collection, animals were deeply anesthetized and blood was collected by cardiac puncture. White adipose tissue was dissected from the gonadal fat pad as previously described [19] and frozen at -80°C until further use. Glucose measurements were immediately made from whole blood using a glucometer. Serum was then isolated and frozen at -20°C until assayed as described below. A second set of females was bilaterally ovariectomized prior to treatment with ritonavir as described above. Briefly, a small incision was made through the abdominal skin and muscle layer of the animal around the area of the kidneys to expose the ovary. The distal portion of each uterine horn was clamped with a hemostat and the ovary was removed.

#### Blood assays

Serum was assayed for metabolic, lipid and hormonal content using enzyme-linked immunoassays (ELISAs). Leptin and insulin were measured using an ELISA from ALPCO Diagnostics (Salem, NH). The intra-assay variance and inter-assay variances for leptin and insulin were 7.8%, 10.5% and 8.7%, 8.5%, respectively. Adiponectin was measured using an ELISA from Linco Research (St. Charles, MO). The intra-assay variance and interassay variances were 5.8% and 6.0%, respectively. Total cholesterol was measured using an assay kit from Biovision (Mountain View, CA). The intra-assay variance and inter-assay variances were 2.8% and 2.6%, respectively. 17β-estradiol was measured with a kit from Research Diagnostic Inc (Concord, MA). The intra-assay variance and inter-assay variances were 4.7% and 7.8%, respectively.

#### Western blot immunoassay

Adipose tissue protein was isolated as previously described [34]. Briefly, proteins were isolated from frozen white adipose tissue by homogenization in 1 mL isolation media (250 mM Sucrose, 0.2 mM EDTA, 10 mM HEPES, ddH_2_0, 1 tablet Roche^® ^Complete Mini protease inhibitor cocktail). The homogenate was then centrifuged at 7000 × g for 30 minutes at 4°C, and the fat pad discarded. After removing the supernatant and pipetting it into a new 1.5 mL tube, the pellet containing the nuclear fraction was brought up in 100–200 μL of sample buffer (40 mM Tris, 2% SDS, pH 8.0, ddH_2_0) and stored at -80°C. 600 μL 10% TCA in acetone + 20 mM DTT was added to the supernatant containing the cytosolic fraction and placed at -20°C for 1–2 hrs. The protein was precipitated by centrifugation at 3500 × g for 30 minutes at 40°C, followed by two washes in ice-cold 90% acetone between which the supernatant was discarded and the pellet precipitated at 3500 × g for 3 minutes at 4°C. After the final wash, the pellet was air dried for 5–10 minutes at room temperature to remove residual acetone. Finally, the cytosolic protein was resuspended in 250–500 μL sample buffer and stored at -80°C. 15 μg of cytosolic protein was separated on a 12.5% SDS-polyacrylamide gel. The separated proteins were then transferred to nitrocellulose membranes. Each membrane was blocked in 1:1 1 × PBS and Odyssey Blocking Buffer (LI-COR) for 1 hour at room temperature. Primary antibodies were diluted in 1:1 1 × PBS and Odyssey Blocking Buffer + 0.2% Tween and incubated overnight at 4°C. The concentrations of the primary antibodies used were: Anti-actin (1:2000, Sigma-Aldrich) and anti-leptin (1:2000, NHPP, NIDDK and Dr. A.J. Parlow). Recombinant leptin was included as a positive control (NHPP, NIDDK, A.J. Parlow). Fluorescently labeled secondary antibodies (Rockland IRDye 800 or Molecular Probes AlexaFluor) were diluted 1:5000 in 1 × PBS + Odyssey Blocking Buffer + 0.2% Tween + 0.01% SDS and incubated with the membrane for 35 minutes in the dark at room temperature. The membrane was then washed and the labeled proteins were visualized on an Odyssey Infrared Imaging System (LI-COR Biosciences, Lincoln, NE) as previously described [35].

#### Statistics

Data were analyzed by two-way analysis of variance (ANOVA), one-way ANOVA, and the Student Newman-Keuls T-test was used for post-hoc comparisons, where appropriate. Significance was considered at a p-value < 0.05. All experiments consisted of n = 6–8 animals per experimental group.

## Competing interests

The author(s) declare that they have no competing interests.

## Authors' contributions

MW conceived the study and participated in its design and coordination. MW wrote the manuscript. KA performed the ELISAs and blood work. EK, DJ and AR treated and monitored the animals and collected food and water data. AB performed the western immunoblot analyses. All authors read and approved the final manuscript.
